# Evidence of Influenza A(H5N1) Spillover Infections in Horses, Mongolia

**DOI:** 10.3201/eid3101.241266

**Published:** 2025-01

**Authors:** Batchuluun Damdinjav, Savitha Raveendran, Laura Mojsiejczuk, Ulaankhuu Ankhanbaatar, Jiayun Yang, Jean-Remy Sadeyen, Munir Iqbal, Daniel R. Perez, Daniela S. Rajao, Andrew Park, Mafalda Viana, Pablo R. Murcia

**Affiliations:** Food and Agriculture Organization of the United Nations, Ulaanbaatar, Mongolia (B. Damdinjav); MRC—University of Glasgow Centre for Virus Research, Glasgow, Scotland, UK (S. Raveendran, L. Mojsiejczuk, P.R. Murcia); State Central Veterinary Laboratory, Ulaanbaatar (U. Ankhanbaatar); The Pirbright Institute, Woking, UK (J. Yang, J.-R. Sadeyen, M. Iqbal); University of Georgia, Athens, Georgia, USA (D.R. Perez, D.S. Rajao, A. Park); University of Glasgow, Glasgow (M. Viana)

**Keywords:** influenza, H5N1, influenza virus, viruses, spillover infections, serology, horses, Mongolia

## Abstract

Recent outbreaks of influenza A(H5N1) have affected many mammal species. We report serologic evidence of H5N1 virus infection in horses in Mongolia. Because H3N8 equine influenza virus is endemic in many countries, horses should be monitored to prevent reassortment between equine and avian influenza viruses with unknown consequences.

Avian influenza viruses (AIVs) of the H5N1 subtype are a cause of concern because they are highly pathogenic in birds and various mammals. H5N1 AIVs have caused outbreaks in both wild and domestic avian species, leading to substantial biodiversity and economic losses from virus-induced deaths and culling interventions. Surveillance studies have shown an increased incidence of H5N1, particularly of clade 2.3.4.4b, in wild birds (*1*), which coincides with growing reports of infections in mammal hosts including skunks, raccoons, bears, and foxes (*2*). In such studies, affected animals were believed to be dead-end hosts, which is consistent with previous perceptions that AIV H5N1 exhibits no or poor transmissibility in mammals. That perception changed in 2022, when outbreaks of H5N1 clade 2.3.4.4b were reported in fur farms in Europe breeding minks and foxes (*3*,*4*) and in populations of pinnipeds (e.g., seals and sea lions) in South America (*5*). In early 2024, an outbreak of AIV caused by genotype B3.13 H5N1, a descendant of H5N1 2.3.4.4b, was reported in dairy cattle in the United States (*6*). At the time, infection was also reported in cats, mice, and farm workers, but direct transmission from cattle could not be confirmed. Cow-to-cow transmission was later confirmed by sequencing data and epidemiologic information.

Horses are natural hosts of equine influenza virus (EIV). Two subtypes of EIV have emerged, including 2 H3N8 strains and 1 H7N7 strain. All EIVs are thought to have originated from AIVs. Here, we report serologic evidence of influenza A(H5N1) infection in horses in Mongolia. 

In surveillance studies during July 2021–October 2023, we collected serum samples from 10 horses from 24 herds, 3 times per year. We recorded associated metadata including sex, approximate age, clinical status, and main use of the horse, as well as location of the herd. Fourteen herds were in the Ugiinuur area of Arkangai Province, a region that exhibits substantial wetlands and hosts a large population of migratory birds. The other 10 herds were in the Dashinchilen area of Bulgan Province and Burd soum of Uvurkhangai, a dry area near the Gobi Desert with low density of wild birds (Figures 1, 2). All horses were unvaccinated and clinically healthy at the time of sampling. The herders reported no history of respiratory disease in the horses. 

**Figure 1:**
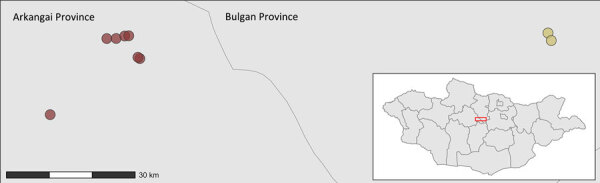
Geographic location of sampling sites for study of influenza A(H5N1) spillover infections in horses, Mongolia. Red represents wetlands and yellow, dry areas. Inset shows location of study area in Mongolia.

**Figure 2:**
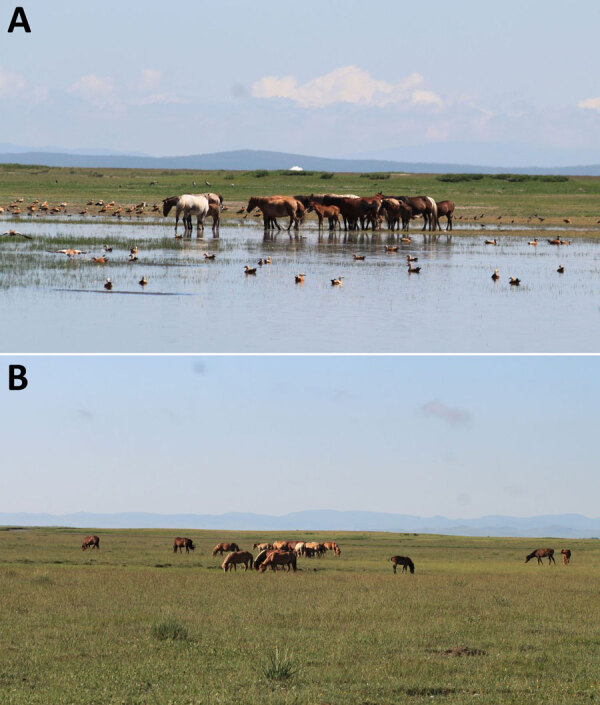
Typical ecosystems of sampling sites for study of influenza A(H5N1) spillover infections in horses, Mongolia. A) Arkangai Province, characterized by large wetlands. B) Bulgan Province, characterized by dry areas.

We heat-inactivated serum samples (n = 2,160), treated them with receptor-destroying enzyme (Denka Company, https://www.denka.co.jp), and performed a screening ELISA assay using an IDEXX influenza A virus antibody test kit (IDEXX Laboratories, https://www.idexx.com) that detects antibodies against IAV nucleoprotein. We further tested nucleoprotein-positive samples (n = 997) for the presence of antibodies against H5 subtype hemagglutinin using an ID Screen Influenza H5 Antibody Competition-FLUACH5 kit (Innovative Diagnostics, https://www.innovative-diagnostics.com); 9 samples were positive, 8 doubtful, and 980 negative. We ruled out cross-reactivity against EIV as a cause of H5 positivity because 13 serum samples from horses experimentally inoculated with different EIV antigens were negative (Appendix). To confirm the H5 ELISA results, we tested all doubtful and positive samples (n = 17) in virus neutralization assays using live virus A/chicken/England/053052/2021, clade 2.3.4.4b (Appendix). Two samples derived from working horses sampled in October 2021 in the Bulgan area, and in October 2022 in Arkangai were positive, with a titer of 1:20. Serum samples from horses experimentally infected with EIV were negative in neutralization assays.

Equids are clearly susceptible to infection by AIV H5N1. Abdel-Moneim et al. (*7*) described an outbreak of influenza in donkeys in Egypt in 2009; they isolated IAV H5N1 from nasal swabs and demonstrated that ≈26% donkeys sampled had been infected. In addition, H5 antibodies were detected in wild asses (*Equus hemonius hemonius*) in Mongolia (*8*); of note, a protein microarray was used in that study and the levels of reactivity were low.

Our findings show that horses are susceptible to infection by H5N1 viruses and that spillover events are likely frequent, highlighting the potential emergence of IAVs by reassortment between H3N8 EIV (the circulating subtype in horses) and H5N1 IAVs. The ecologic conditions for reassortment are met in North America; 30% of the global horse population is located (*9*) there, EIV is endemic, avian influenza A(H5N1) clade B3.13 is spreading in cattle, and contact rates between cows and horses are likely to be high in agricultural settings. Consistent with our previous work showing that horses in Mongolia are commonly exposed to H3N8 AIVs in the absence of disease outbreaks (*10*), our results suggest that H5N1 infections in horses are likely to be subclinical, posing challenges to virus detection. We recommend serologic surveys in premises that keep horses; such studies would aid early virus detection, provide a comprehensive picture of the changing ecology of IAVs, and inform the design of control measures to prevent influenza emergence.

**Appendix.** Additional information about H5N1 influenza spillover infections in horses, Mongolia.
