# Supplementary material for: Evidence of Influenza A(H5N1) Spillover Infections in Horses, Mongolia
Source: Emerg Infect Dis. 2025 Jan;31(1):183–5. doi: 10.3201/eid3101.241266 (PMC11682804; doi:10.3201/eid3101.241266)
Supplement: Supplementary file 1 — Appendix. Additional information about H5N1 influenza spillover infections in horses, Mongolia. [file 24-1266-Techapp-s1.pdf]

Article DOI: https://doi.org/10.3201/eid3101.241266

EID cannot ensure accessibility for supplementary materials supplied by authors. Readers who have difficulty accessing supplementary content should contact the authors for assistance.

Evidence of Influenza A(H5N1) Spillover Infections in Horses, Mongolia

Appendix

H5 ELISA Controls

H5 antibodies were detected using the ID Screen Influenza H5 Antibody Competition-FLUACH5 kit (IDVet) as described in the main text. This is a competitive ELISA. The manufacturer's instructions were followed without introducing any modifications. To validate the assay, 13 serum samples obtained from horses experimentally inoculated with the following H3N8 equine influenza viruses (EIV) were tested: A/equine/La Plata/1993; A/equine/Kentucky/91; A/equine/Kentucky/99; A/equine/Kentucky/5/2002; A/equine/Ohio/2003; A/equine/South Africa/4/2003; A/equine/Ibaraki/1/2007; A/equine/Kentucky /7/2007; A/equine/Richmond/1/2007; A/equine/Yokohama/aq13/2010; A/equine/Kentucky /1/2014; A/equine/Yokohama/aq100/2017; A/equine/Arizona (AZ)/2019. Serum from a negative control horse (naive) was also included. All samples were negative for H5 and positive for NP. These results show that the ELISA approach used to detect anti-H5 antibodies does not cross-react with equine antisera positive to H3N8 equine influenza viruses.

Confirmation of ELISA Results

A virus neutralization assay was used as a second confirmatory test. This assay was performed in high containment laboratories using live virus (A/chicken/England/053052/2021, H5N1, clade 2.3.4.4b). Chicken antiserum raised against A/chicken/England/053052/2021 was used as a positive control. Naive chicken antiserum was used as a negative control. Additional controls for cross-reactivity included serum samples from horses experimentally infected with

A/equine/La Plata/1993, A/equine/Kentucky/5/2002, and A/equine/Yokohama/aq100/2017. An additional negative control was a serum sample from a naive horse. Briefly, serum samples were heat-inactivated at 56oC for 30 min, and then diluted 1:20 in DMEM. The serum samples were incubated with 100 PFU of A/chicken/England/053052/2021at 37oC 1hour. Monolayered MDCK cells in 96-well plates were washed with PBS three times and then infected with virus-serum mixture for 1 hour at 37oC. Cells were then washed with PBS three times and maintained in DMEM for three days. After three days post infection, the cells were stained with crystal violet for visualisation.
